# ^18^ F-Fluoride positron emission tomography/computed tomography for noninvasive *in vivo* quantification of pathophysiological bone metabolism in experimental murine arthritis

**DOI:** 10.1186/ar4670

**Published:** 2014-07-22

**Authors:** Ingo M Irmler, Peter Gebhardt, Bianca Hoffmann, Thomas Opfermann, Marc-Thilo Figge, Hans P Saluz, Thomas Kamradt

**Affiliations:** 1 Institute of Immunology, Jena University Hospital, Leutragraben 3, Jena 07743, Germany; 2 Leibniz Institute for Natural Product Research and Infection Biology, Hans-Knöll-Institute, Beutenbergstrasse 11a, Jena 07745, Germany; 3 Department of Nuclear Medicine, Jena University Hospital, Bachstrasse 18, Jena 07743, Germany; 4 Friedrich Schiller University, Fürstengraben 1, Jena 07743, Germany

## Abstract

**Introduction:**

Evaluation of disease severity in experimental models of rheumatoid arthritis is inevitably associated with assessment of structural bone damage. A noninvasive imaging technology allowing objective quantification of pathophysiological alterations of bone structure in rodents could substantially extend the methods used to date in preclinical arthritis research for staging of autoimmune disease severity or efficacy of therapeutical intervention. Sodium ^18^ F-fluoride (^18^ F-NaF) is a bone-seeking tracer well-suited for molecular imaging. Therefore, we systematically examined the use of ^18^ F-NaF positron emission tomography/computed tomography (PET/CT) in mice with glucose-6-phosphate isomerase (G6PI)–induced arthritis for quantification of pathological bone metabolism.

**Methods:**

F-fluoride was injected into mice before disease onset and at various time points of progressing experimental arthritis. Radioisotope accumulation in joints in the fore- and hindpaws was analyzed by PET measurements. For validation of bone metabolism quantified by ^18^ F-fluoride PET, bone surface parameters of high-resolution μCT measurements were used.

**Results:**

Before clinical arthritis onset, no distinct accumulation of ^18^ F-fluoride was detectable in the fore- and hindlimbs of mice immunized with G6PI. In the course of experimental autoimmune disease, ^18^ F-fluoride bone uptake was increased at sites of enhanced bone metabolism caused by pathophysiological processes of autoimmune disease. Moreover, ^18^ F-fluoride signaling at different stages of G6PI-induced arthritis was significantly correlated with the degree of bone destruction. CT enabled identification of exact localization of ^18^ F-fluoride signaling in bone and soft tissue.

**Conclusions:**

The results of this study suggest that small-animal PET/CT using ^18^ F-fluoride as a tracer is a feasible method for quantitative assessment of pathophysiological bone metabolism in experimental arthritis. Furthermore, the possibility to perform repeated noninvasive measurements *in vivo* allows longitudinal study of therapeutical intervention monitoring.

## Introduction

Rheumatoid arthritis (RA) is one of the most common autoimmune diseases, affecting approximately 1% of the population in Europe and North America. Bone erosion can be detected as early as several weeks after the onset of the first clinical signs and symptoms of RA [1]. Pathogenesis in preclinical models is similar to that in clinical RA; it is characterized by inflammation and bone destruction. In the past few decades, histopathological evaluation of joint sections has mainly been used for assessment of inflammation and bone destruction in small-rodent models of arthritis. Recently, we showed the benefit of state-of-the-art imaging modalities for visualization and quantitative assessment of inflammation in glucose-6-phosphate isomerase (G6PI)–induced arthritis using 2-deoxy-2-^18^ F-fluoroglucose (^18^ F-FDG) positron emission tomography/computed tomography (PET/CT) [2]. Also, it has been shown that cell proliferation can be detected in experimental arthritis with the PET proliferation tracer 3′-deoxy-3′-^18^ F-fluorothymidine [3].

^18^ F-fluoride not only can be used to label glucose and other molecules of physiologic relevance but also has favorable properties in the form of sodium ^18^ F-fluoride (^18^ F-NaF) as a radiotracer *per se* in noninvasive *in vivo* imaging to investigate musculoskeletal diseases [4]. The use of ^18^ F-NaF as a bone imaging probe was established by Blau *et al*. in the early 1960s [5], but it was subsequently replaced by ^99m^Tc-labeled tracers due to their availability, lower costs and the lower energy of 140-keV photons, allowing the use of γ-cameras. ^18^ F-fluoride PET is an increasingly used molecular imaging modality, not only in human skeletal disorders but also in small-animal preclinical research [6-8]. This is facilitated by the distribution and use of three-dimensional PET scanners with high spatial resolution in clinical medicine and advantages over ^99m^Tc-labeled bone agents used for skeletal scintigraphy, such as higher diagnostic accuracy and reduced scanning time. Compared to γ-cameras, molecular imaging using PET provides the advantages of higher spatial resolution, higher sensitivity and three-dimensional tomographic image reconstruction. Furthermore, the combination of PET with μCT enables attenuation correction of radiotracer signaling, allowing quantitative measurements using ^18^ F-fluoride PET/CT.

Applied ^18^ F-NaF, dissociated into Na^+^ and ^18^ F^−^, is rapidly cleared from the blood and accumulates in the bone, where, on the hydroxyapatite surface, an OH^−^ ion is replaced by an ^18^ F^−^ ion to form fluorapatite. The incorporation of ^18^ F-fluoride in the bone is determined by vascular perfusion and bone surface accessible for ion exchange, indirectly reflecting bone formation and bone resorption [9]. This means that ^18^ F-NaF can be used not only for the common measurement of bone mineral deposition but also for visualization of osteolytic increases of exposed bone surface, such as in the context of musculoskeletal autoimmune disease [10].

In clinical oncology, primary bone tumors and skeletal metastasis can reliably be detected by ^18^ F-fluoride PET [4,11]. In mice, pathological osteoblastic activity can be detected even earlier by ^18^ F-fluoride PET/CT imaging than by radiography and corresponds to histological evaluation of increased bone formation [7]. As with bone tumor pathogenesis, a pathologically increased bone metabolism is a central feature of chronic arthritis, resulting in functional disorders of the joints [12]. Therefore, in our present study, we examined the use of ^18^ F-NaF small-animal PET/CT for the quantitative and noninvasive *in vivo* assessment of pathophysiological bone metabolism in acute and chronic stages of experimental G6PI-induced murine arthritis. ^18^ F-NaF PET quantification of bone destruction, visible as lesions in cortical bone surface and dysregulated bone formation, was validated using high-resolution CT measurements of the paws.

## Methods

### Glucose-6-phosphate isomerase–induced arthritis

DBA/1 mice were bred at the animal facility of the Jena University Hospital (Jena, Germany). All animal studies were approved by the local commission for animal protection (Thüringer Landesamt für Verbraucherschutz, Bad Langensalza, Germany; registered number 02-045/08). Arthritis was induced as described elsewhere [13]. In brief, DBA/1 mice were immunized subcutaneously with 400 μg of recombinant human G6PI in emulsified complete Freund’s adjuvant (Sigma-Aldrich, Taufkirchen, Germany). Macroscopic evaluation of arthritis was performed according to severity of clinical manifestations in wrist and ankle joints, in metacarpophalangeal and metatarsophalangeal joints, and in digits and toes. Swelling and redness in wrist and ankle joints and in metacarpophalangeal and metatarsophalangeal joints, respectively, were graded from 0 to 3. A score of 0 indicates no macroscopically recognizable signs of arthritis, 1 indicates swelling and redness, 2 means strong swelling and redness and 3 indicates massive swelling and redness. Additionally, the number of digits and toes with inflamed joints were divided in half to avoid assessment imbalance because inflammation in the G6PI-induced arthritis model is located mainly in proximal joints of the paws [2]. To calculate the total clinical score per animal, results from all paws were summed. For PET/CT measurements, mice were anesthetized with 1.5% to 2% isoflurane (Deltaselect, Dreieich, Germany) vaporized in oxygen (1.5 L/min) to prevent animal movement and reduce imaging artifacts. Respiration of mice under anesthesia was monitored. ^18^ F-fluoride (half-life = 109 minutes; Eckert & Ziegler, Bad Berka, Germany) with an activity of 10.4 ± 0.8 MBq was injected intravenously into the lateral tail vein. Longitudinal arthritis imaging was performed at various time points of acute and chronic clinical arthritis (*n* = 3 to 6 mice per time point). We obtained dynamic PET scans for kinetic analysis of tracer uptake in nonimmunized mice (*n* = 3).

### Positron emission tomography/computed tomography *in vivo* imaging

*In vivo*^18^ F-NaF imaging was performed using a Siemens Inveon small-animal multimodality PET/CT system (Preclinical Solutions, Siemens Healthcare Molecular Imaging, Knoxville, TN, USA), characterized by the combination of two independently operating PET and CT scanners. Radial, tangential and axial resolutions at the center of the field of view of the PET module are 1.5 mm for this imaging modality [14,15]. PET image acquisition was carried out with a coincidence timing window of 3.4 ns and an energy window of 350 to 650 keV. PET data acquisition was performed for 1,800 seconds, starting 50 minutes after tracer application. In kinetic analysis, PET data acquisition lasted 5,400 seconds and started concomitantly with radiotracer injection. Images were reconstructed into three-dimensional images using Fourier rebinning and three-dimensional ordered-subset expectation maximization algorithm. Attenuation of PET data was corrected based on the CT measurements. The CT module consists of a cone-beam X-ray μCT source (50-μm focal spot size) and a 3,072 × 2,048–pixel X-ray detector. In our μCT imaging protocol, we used an axial–transaxial resolution of 2,048 × 2,048–pixel, 80 kVp at 500 μA, 360° rotation and 360 projections per bed position for paw measurements and 3,072 × 2,048–pixel, 220° rotation and 120 projections per bed position for whole-animal attenuation scans, respectively. CT images were reconstructed using a Shepp-Logan filter and cone-beam–filtered back projection. To reduce stress due to overly prolonged measurement times, only high-resolution μCT data from hindpaws were acquired for correlation analysis of bone surface assessment with ^18^ F-fluoride PET and CT.

### Assessment of pathophysiological bone metabolism with ^18^ F-fluoride or μCT

Quantitative analysis of ^18^ F-fluoride accumulation in the period from 50 to 80 minutes after ^18^ F-fluoride injection or 0 to 90 minutes, respectively, was enabled by image fusion technology in Siemens Inveon Research Workplace 4.0 software. For dynamic PET analysis, scans were started 5 seconds before radiotracer injection and continued for 90 minutes. The 90-minute data set was divided into 45 time frames (6 × 10 seconds, 6 × 20 seconds, 7 × 60 seconds, 10 × 120 seconds, 10 × 180 seconds and 6 × 300 seconds) during histogramming to construct time–activity curves. For static analysis, data from 75 to 80 minutes after ^18^ F-fluoride injection were analyzed in a single time frame. Radioisotope activity in the venous blood pool or in fore- or hindpaws, reflecting bone incorporation of ^18^ F-fluoride, was measured as standardized uptake value (SUV; g/ml) using PMOD 3.15 software (PMOD Technologies Ltd, Zurich, Switzerland) or Siemens Inveon Research Workplace 4.0 software. Guided by maximum intensity projection images, volumes of interest were drawn as spheres (ellipsoids) over anatomic structures of bones and joints. A threshold of 40% (max–min) was used to separate the site of tracer accumulation from background tissue signaling. Visualization of skeletal elements of the hindpaws was also done using PMOD 3.15 software.

Analyses of metatarsal CT data were performed using Definiens Developer XD™ 2.0.3 build 2015 software (Definiens, Munich, Germany). For segmentation of metatarsal bones, the automatic threshold routine was used. Then, individual bones were discriminated by watershed segmentation. Oversegmented fragments were merged manually to achieve three-dimensional objects of each bone. After identification of metatarsals 1 to 5, a local threshold was applied on each metatarsal bone to refine bone margins and remove remaining nonbone pixels. The threshold was calculated as the difference between mean pixel gray value and standard deviation of pixel gray values. Quantitative measurements of bone surface and bone volume were calculated for each metatarsal bone.

### Statistical analysis

Statistical differences between groups were evaluated using the nonparametric Mann-Whitney *U* test. Correlation analyses (Spearman test) were performed for PET data and clinical scoring or PET and CT data, respectively, whereas CT surface data of all metatarsals, per paw, were summed. Statistical significance was accepted at *P* < 0.05 (*P < 0.05, **P < 0.01, ***P < 0.001). In bar charts and text, data are given as arithmetic means and standard errors of the mean (SEM). All calculations were performed using the software package IBM SPSS Statistics version 20.0 (IBM, Ehningen, Germany).

## Results

### Inflammation and bone destruction in glucose-6-phosphate isomerase–induced arthritis

**Figure 1:**
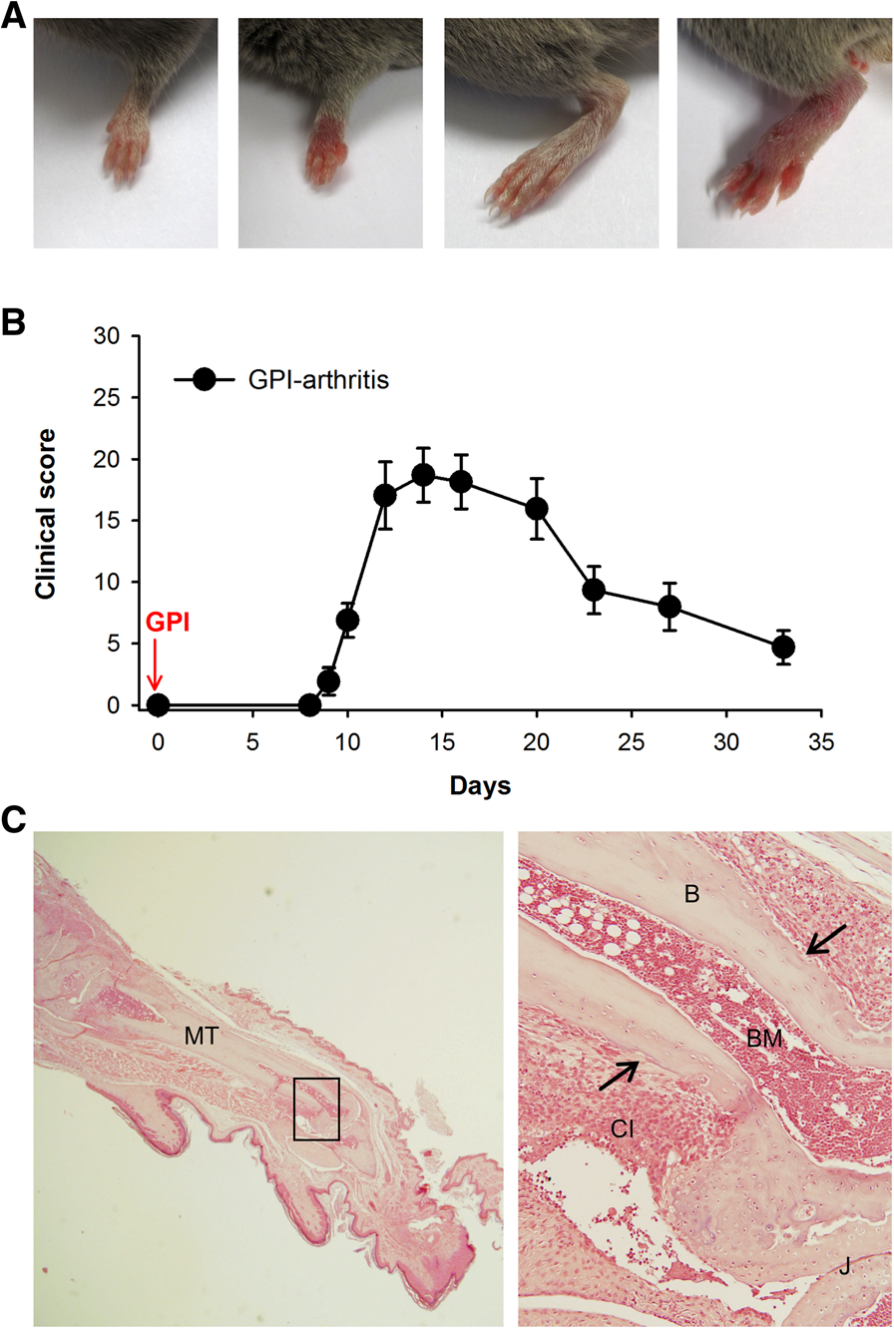
**Joint inflammation in glucose-6-phosphate isomerase–induced arthritis. (A)** Photographs showing inflammation in fore- and hindpaws of arthritic mice (two at right) and nonimmunized control animals (two at left). **(B)** Clinical course of inflammatory arthritis in DBA/1 mice (*n* = 7). GPI, Glucose-6-phosphate isomerase induction. **(C)** Arthritis was associated with destruction of bone. Shown is a representative image of metatarsal bone revealing cortical bone lesions (arrows) and infiltration of immune cells to the sites of inflammation. B, Bone; BM, Bone marrow; J, Joint; CI, Cellular infiltrate; MT, Metatarsal. Original magnification = 20× (left) and 200× (right).

Immunization of animals with G6PI induced severe paw inflammation in DBA/1 mice (Figure 1A). Experimental disease was characterized by redness and swelling in wrist and ankle joints, as well as in dorsal areas of the paws and in metatarsophalangeal, metacarpophalangeal and phalangeal joints. Macroscopic scoring revealed onset of polyarthritis around 9 days after disease induction, maximum of inflammation at day 14 (d14) and a subsequent decrease until d33 (Figure 1B). Histopathology revealed bone erosion in skeletal elements of the inflamed paws (Figure 1C).

### Kinetics of ^18^ F-fluoride bone uptake in mice

**Figure 2:**
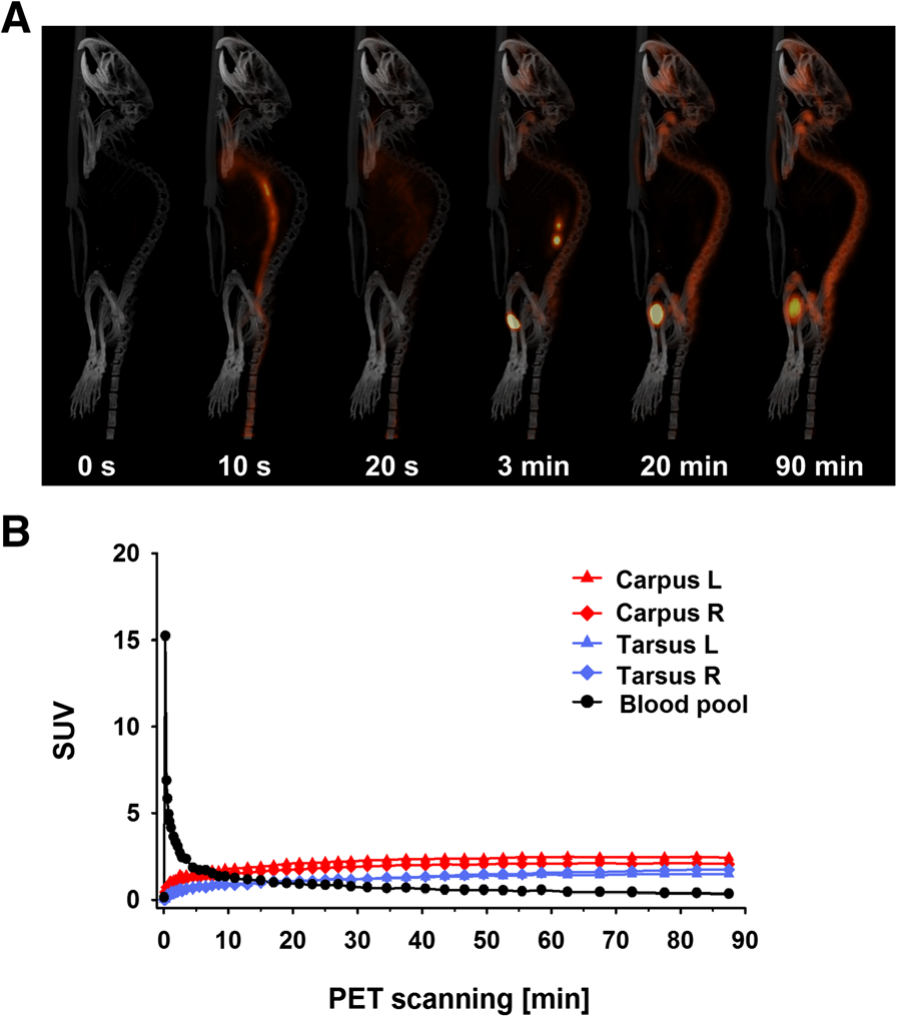
**Biodistribution and bone accumulation of **^**18**^ **F-fluoride in naïve mice. (A)** Positron emission tomography (PET) scans (maximum intensity projection) of a naïve mouse after intravenous ^18^ F-fluoride tail vein injection. Radiotracer was immediately distributed in the whole organism via the circulation and rapidly accumulated in the skeletal elements. **(B)** The 90-minute ^18^ F-fluoride time–activity curve reveals rapid ^18^ F-fluoride clearance from the bloodstream (via the venae cavae) and subsequent ^18^ F-fluoride accumulation in the bones of the fore- and hindpaws. SUV, Standardized uptake value.

Radiotracer applied intravenously in the tail vein was immediately (0 to 10 seconds postinjection) transported to the heart via the blood flow (Figure 2A). Subsequent whole-body distribution of ^18^ F-fluoride resulted in thoracic and intestinal PET signaling (10 to 20 seconds postinjection). After 3 minutes, the pattern of ^18^ F-fluoride PET signaling indicated onset of bone accumulation, and signaling from the kidneys and bladder revealed partial radiotracer excretion. Twenty minutes after radiotracer injection, signaling of nonexcreted ^18^ F-fluoride was restricted mainly to bone and continued to increase until the end of measurement at 90 minutes.

Concomitantly, the quantitative time–activity curve analysis of ^18^ F-fluoride paw uptake revealed rapid radiotracer accumulation beginning immediately after injection and tracer signaling remaining at almost the same level from 40 to 90 minutes postinjection (Figure 2B). At 45 minutes postinjection, 90% of ^18^ F-fluoride uptake was achieved (SUV_mean_ = 1.8), compared to bone radiotracer signaling after 90 minutes (SUV_mean_ = 1.9). In contrast, blood pool tracer activity measured in the *venae cavae* region showed a rapid decline after an activity maximum (SUV_max_ = 15.2), within minutes following injection, reflecting rapid clearance of ^18^ F-fluoride from the blood.

### Localization of exact sites of ^18^ F-fluoride paw bone uptake in glucose-6-phosphate isomerase–induced arthritis

**Figure 3:**
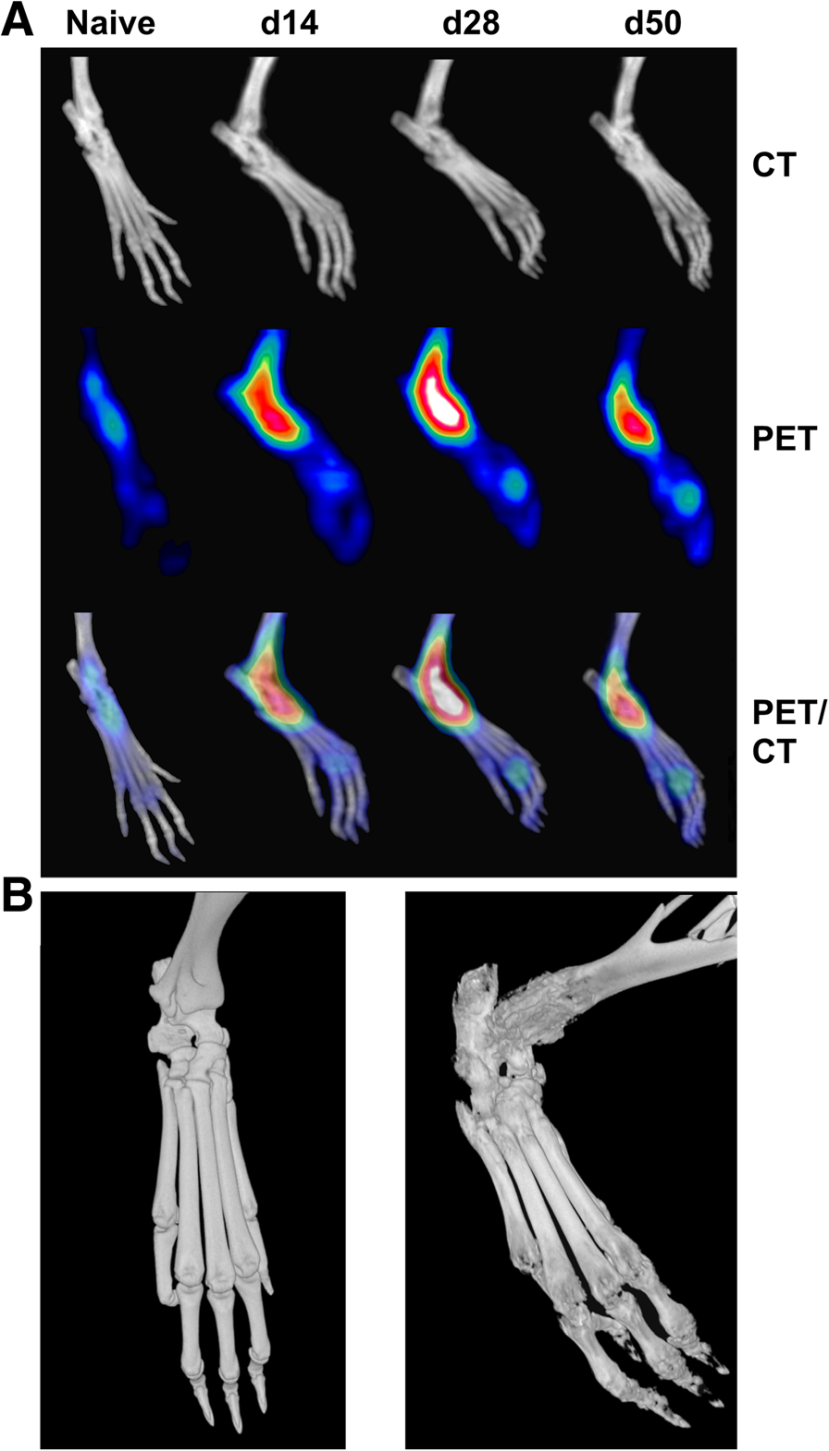
**Localization of peripheral **^**18**^ **F-fluoride accumulation in glucose-6-phosphate isomerase–induced arthritis. (A)** In naive mice, administration of ^18^ F-fluoride resulted in low bone tracer accumulation in the subtalar joints. Under acute arthritic conditions (day 14 (d14)), there was distinct radiotracer signaling at sites of manifested disease, whereas increased ^18^ F-fluoride uptake was detectable in chronic arthritis (d28). In late remitting arthritis (d50), ^18^ F-fluoride uptake declined. Shown are representative images of murine hindpaws obtained with micro–computed tomography (μCT; top), ^18^ F-fluoride positron emission tomography (PET; middle) and coregistered PET and CT (bottom). **(B)** High-resolution μCT images of hindpaws obtained before arthritis onset (left) and with chronic arthritis (right) reveal the impact of inflammatory autoimmune disease on bone and joint integrity.

Detailed three-dimensional anatomical μCT measurements allowed identification of metatarsal and phalangeal bones and joints, and PET imaging revealed radiotracer uptake in the paws during the course of experimental arthritis (Figure 3A). Low ^18^ F-fluoride accumulation before immunization switched to a distinct tracer uptake in the acute (d14) and chronic (d28) stages of disease and declined during late remitting arthritis (d50). Coregistration of PET and CT data enabled exact localization of ^18^ F-fluoride accumulation in arthritic mice.Detailed, three-dimensional anatomical information obtained by high-resolution μCT enabled visualization of the bone surfaces of phalangeal, metatarsal, tarsal and tibial bones. Compared to nonarthritic control animals (Figure 3B, left), pathologic bone metabolism in the mice with G6PI-induced arthritis induced a distinct increase in bone roughness (Figure 3B, right).

### Quantification of pathophysiological bone metabolism using ^18^ F-fluoride PET in progressing murine arthritis

**Figure 4:**
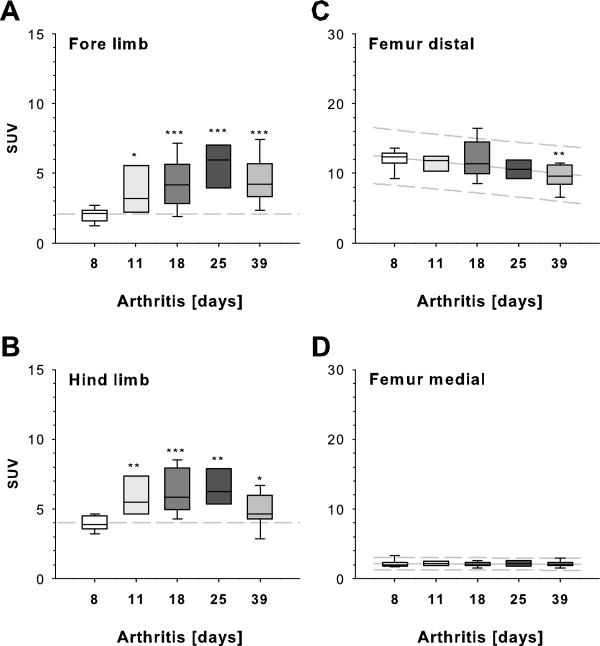
**Quantification of **^**18**^ **F-fluoride uptake in progressive arthritis.** Pathological effects in arthritic bone metabolism differed from ^18^ F-fluoride accumulation before clinical arthritis onset can be seen above an arbitrary baseline of 2 and 4 standardized uptake value (SUV) for fore and hindpaws, respectively. **(A)** Two days after onset of clinical arthritis at day 9, uptake of ^18^ F-fluoride in forelimbs was significantly enhanced (day 11). In chronic arthritis at days 18 and 25, ^18^ F-fluoride accumulation was further increased significantly compared to day 8, whereas in late chronic arthritis (day 39), tracer uptake was declining (*n* = 6 to 12 paws per time point). **(B)** Hindlimb pathological arthritic bone metabolism, reflected by ^18^ F-fluoride signaling in the tarsal joint and metatarsal bones, coincided with forelimb ^18^ F-fluoride signaling (*n* = 6 to 12 paws per time point). **(C)** High-level ^18^ F-fluoride uptake in distal femoral bone was similar at various time points in the course of arthritis pathogenesis, except for a significant decrease at day 39. **(D)** Low-level ^18^ F-fluoride uptake in medial cortical femoral bone was comparable at various time points of arthritis pathogenesis. *P < 0.05, **P < 0.01, ***P < 0.001.

To analyze the usability of the ^18^ F-fluoride PET imaging modality for quantification of pathological bone metabolism in experimental arthritis models, we measured ^18^ F-fluoride uptake in all paws of mice with G6PI-induced arthritis at five different time points of disease. Before onset of clinical arthritis (that is, in bone tissue without pathological changes (d8), there were only low levels of ^18^ F-fluoride accumulation in wrist (Figure 4A) and ankle (Figure 4B) joints, measured as SUV. In acute clinical arthritis at d11, paw inflammation was associated with an increase in bone metabolism and induced a significant increase of ^18^ F-fluoride uptake compared to d8 (*P* = 0.019 (wrist) and *P* = 0.002 (ankle)). Mean SUVs at d8 were 2.08 ± 0.1 in carpal joints and 4.0 ± 0.2 in tarsal joints, and at d11 mean SUVs were 3.8 ± 0.6 in carpal joints and 5.8 ± 0.5 in tarsal joints. Mean ^18^ F-fluoride uptake was further significantly increased at d18 (4.4 ± 0.5 (carpal) and 6.3 ± 0.5 (tarsal)) and d25 (5.6 ± 0.6 (carpal) and 6.5 ± 0.6 (tarsal)), followed by a decrease at d39 (4.4 ± 0.5 (carpal) and 5.0 ± 0.4 (tarsal)). PET data revealed a 1.3- to 2.8-fold increase in ^18^ F-fluoride bone uptake in the paws at progressive stages of inflammatory arthritis, which always differed significantly from ^18^ F-fluoride accumulation before onset of clinical disease. This finding indicated a rise of pathological bone-degrading and bone-forming processes leading to joint dysfunction, characteristic for RA and its animal models.

To examine the general effects of arthritis on ^18^ F-fluoride uptake in elements of the skeleton, we quantified PET signaling in medial and distal femoral bones. SUVs revealed high tracer uptake (SUV > 10) in trabecular distal areas of the femur (Figure 4C). Except for late remitting arthritis (d39), SUVs remained at a similar level at different stages of G6PI-induced arthritis, indicating only a slight influence of pathological bone metabolism associated with arthritis on distal femoral tracer uptake. In contrast to trabecular distal areas, mean SUVs in median cortical areas of the femur ranged from 2.1 to 2.2 in all stages of arthritis (Figure 4D), additionally negating general effects of arthritis on bone accumulation of ^18^ F-fluoride at sites without clinical manifestations of disease. ^18^ F-fluoride PET/CT measurements in healthy mice without any pathological conditions revealed a nonequal pattern of bone tracer uptake (Additional file 1: Figures S1A and S1B). There were hotspots of PET signaling in proximal joints of the pectoral girdle; in the pelvis, including the knees; and in spinal and cranial bones. Volume-rendering of the knee joints revealed a coincidence of increased ^18^ F-fluoride accumulation and trabecular bone (Additional file 1: Figure S1C). Therefore, we assume that the increased surface in trabecular bones, meeting the demands of distinct mechanical stress or saving weight, is the cause for the observed heterogeneous pattern of ^18^ F-fluoride uptake in skeletal bone.

### Quantification of pathophysiological bone metabolism using μCT

**Figure 5:**
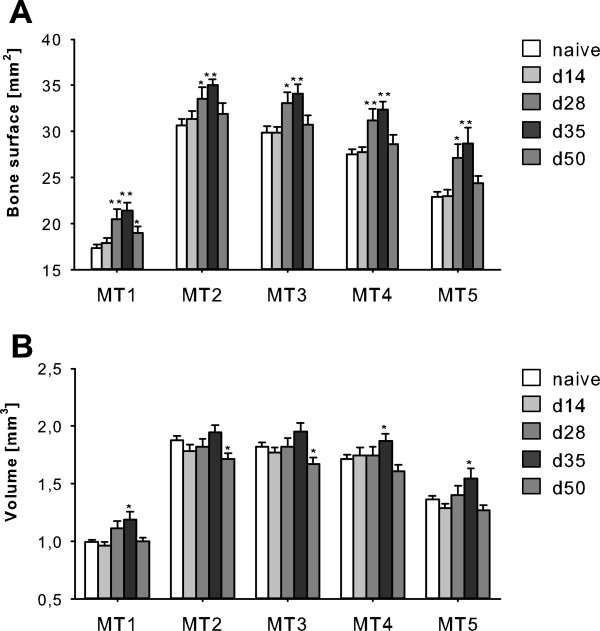
**Quantification bone parameters on high-resolution μCT images. (A)** Quantification of the surface of all five metatarsal bones (MT1 to MT5) revealed no difference in bone structure between naïve mice and those with acute arthritis (day 14 (d14)), when inflammation was at its maximum. In contrast, bone surface area was significantly increased in chronic arthritis (d28 and d35). In late chronic arthritis (d50), surface area declined to preinflammation levels. **(B)** Volumes of metatarsal bones were almost unaffected by disease pathogenesis, except for significant increases in MT1, MT4 and MT5 at d28 and significant decreases in MT2 and MT3 at d50 (*n* = 6 to 12 paws per time point). *P < 0.05, **P < 0.01, ***P < 0.001.

To validate assessment of pathophysiological bone metabolism by ^18^ F-fluoride PET imaging, bone parameters in μCT data were quantified. Bone surface showed no difference for metatarsals 1 to 5 between unimmunized mice and mice with acute inflammatory arthritis (Figure 5A, d14). In chronic and remitting arthritis (Figure 5A, d28, d35 and d50), bone surface was significantly increased due to bone lesions or dysfunctional bone formation. The course of bone surface assessment was consistent with the course of SUV data obtained by ^18^ F-fluoride PET. The volume of metatarsal bones revealed a slight increase at d35, followed by a subsequent decline to previous levels in late experimental arthritis at d50 (Figure 5B). Overall, bone volume was largely unaffected by arthritis pathogenesis.

### Regression analysis of clinical parameters and quantitative PET and CT assessment of bone parameters

**Figure 6:**
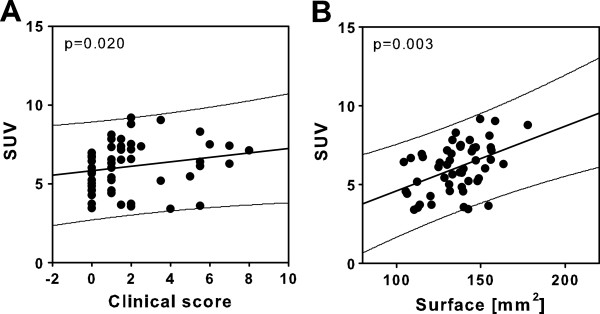
**Correlation analysis of arthritis assessment by **^**18**^ **F-fluoride and μCT imaging. (A)** Correlation analysis of quantitative ^18^ F-fluoride positron emission tomography/computed tomography (PET/CT) hindpaw measurements and semiquantitative macroscopic scoring of paws at different stages of glucose-6-phosphate isomerase (G6PI)–induced arthritis demonstrated a significant correlation of both parameters. SUV, Standardized uptake value. **(B)** Quantitative assessment of ^18^ F-fluoride paw uptake PET and the sum of surface of metatarsal bones 1 to 5 according to μCT data were significantly correlated (*n* = 60 paws). *P < 0.05, **P < 0.01, ***P < 0.001.

To validate quantification of pathological bone metabolism by ^18^ F-fluoride PET imaging in experimental arthritis, we performed correlation analysis of quantitative PET results with semiquantitative clinical scoring data and assessment of bone surface roughness destruction by μCT. Although inflammation and bone destruction are associated but functionally not necessarily constrained mechanisms of arthritis pathophysiology, macroscopic clinical scoring and SUV of ^18^ F-fluoride were significantly correlated (Figure 6A). More importantly, there was a significant correlation of quantitative assessment of pathological bone metabolism by ^18^ F-fluoride SUV and assessment of bone surface by μCT (Figure 6B), demonstrating the feasibility of these modalities for quantification of bone destruction in experimental arthritis. Therefore, small-animal ^18^ F-fluoride PET/CT is a reliable imaging method for *in vivo* quantification of arthritis-induced bone erosion and bone malformation, correlating with assessment of pathophysiologic bone surface alterations using μCT.

## Discussion

The usefulness of the small-animal PET/CT imaging modality using ^18^ F-NaF as a radiotracer is not restricted to the detection of primary bone tumors and skeletal metastasis in cancer research. ^18^ F-fluoride PET/CT imaging is also a valid technique for use in the assessment of disease severity according to pathological bone turnover in the field of preclinical arthritis research. In this study, in which we employed the G6PI-induced arthritis model, we focused on the feasibility of using ^18^ F-fluoride PET to visualize and quantify pathological bone metabolism in distal murine arthritic joints noninvasively and *in vivo*. In addition to joint inflammation, which can easily be quantified by ^18^ F-FDG PET/CT according to activation and proliferation of resident cells and migrated cells of the immune system, bone damage is the second major parameter used for assessment of arthritis severity [2].

In contrast to ^18^ F-FDG, which is trapped in cells at sites of inflammation due to pathologically increased glucose metabolism, ^18^ F-fluoride represents for specific radiotracer accumulation in the bone. Erosive processes degrading bone and cartilage and dysfunctional bone repair mechanisms in RA and its animal models are associated with an increased bone surface. Therefore, the increased mineral-binding capacity results in site-specific ^18^ F-fluoride uptake in arthritic joints, which can easily be used for visualization and, more importantly, provides a measurement method for the quantification of pathological bone metabolism in preclinical arthritis models. The results of our studies show increased uptake of ^18^ F-fluoride in the paws of arthritic mice and reveal that the ^18^ F-fluoride PET/CT quantification of pathologic bone metabolism in arthritis pathogenesis significantly correlated with our assessment of pathophysiologic bone surface alterations based on high-resolution μCT measurements.

Radioisotope imaging is, to date, one of the most applicable imaging modalities for functional and whole-body *in vivo* quantification of bone metabolism in mice. Compared to other radiopharmaceuticals used for bone imaging, such as ^99m^Tc, ^18^ F-fluoride has some beneficial attributes. First, ^18^ F-fluoride has a high affinity to bone, resulting in favorable skeletal kinetics. Within 60 minutes after intravenous injection, only 10% of the injected dose is still located in the bloodstream [10]. Thus the concurrence of rapid bone uptake and fast blood-pool clearance yields a favorable bone-to-background ratio. Additionally, ^18^ F-fluoride does not accumulate in inflamed soft tissue and only minimally binds to serum proteins [16]. Furthermore, small-animal ^18^ F-NaF PET measurements have an excellent reproducibility in animal models of bone disease [6,17].

One limiting factor in ^18^ F-fluoride PET imaging might be vascularization of the tissue restricting tracer delivery. In contrast to epithelial tissue, the circulation in well-vascularized bone tissue is less affected by exogenous factors, allowing reproducible data acquisition. In experimental arthritis, the increased vascularization and blood flow in inflamed tissue may influence tracer delivery and, therefore, PET signaling at stages of acute inflammation. Nevertheless, at time points of maximal tracer uptake in our experiments, clinical arthritis was already remitting and macroscopically visible signs of inflammation were diminished. Another important aspect of ^18^ F-fluoride PET imaging is bone structure. Skeletal elements consisting of cortical and trabecular bone result in strong baseline PET signaling. This is presumably caused by the manifold increase in bone surface and less by increased metabolism at these sites, as binding capacities of cortical and trabecular bone are only slightly different. However, both decrease significantly if bone matrix is demineralized [18]. Therefore, if bones with cancellous and noncancellous structures are located near regions of interest, data analyses require a high degree of anatomical accuracy, which can be achieved with μCT. In quantification of experimental arthritis severity, we found that the effect of high baseline signaling in tibial and radial bones was of negligible relevance for ^18^ F-fluoride PET imaging, because signaling hotspots of arthritis pathogenesis were located in tarsal, metatarsal and carpal and metacarpal bones. Another source of a high and pathological ^18^ F-fluoride baseline PET signaling independent of autoimmune disease pathogenesis might be the preoccurrence of osteoarthritis resulting in mechanical bone erosion. Whereas this aspect is not relevant for imaging in experimental arthritis models, it should be considered in RA bone imaging, where age is a risk factor.

## Conclusion

In our present study, we demonstrate the capability of ^18^ F-fluoride PET to monitor and quantify pathological bone conditions in the model of G6PI-induced arthritis. Furthermore, we validated this bone imaging technique successfully by using bone surface μCT data. Because ^18^ F-fluoride PET is a noninvasive and nondestructive way to measure bone metabolism *in vivo*, this molecular imaging modality is not only useful for numerous applications in basic animal science, where pathophysiological bone metabolism is of interest, but also is a valuable tool for use in preclinical arthritis research, where pathological bone destruction and inflammation are the major parameter used for assessment of disease severity.

## Abbreviations

CT: Computed tomography; ^18^ F-FDG: ^18^ F-labelled fluorodeoxyglucose; G6PI: Glucose-6-phosphate isomerase–induced arthritis; MT: Metatarsal bone; PET: Positron emission tomography; RA: Rheumatoid arthritis; SUV: Standardized uptake value.

## Competing interests

The authors declare that they have no competing interests.

## Authors’ contributions

II was responsible for study conception and design, animal experiments and data collection, PET data analysis and interpretation and manuscript writing. PG was responsible for study design, animal experiments and data collection, PET data analysis and manuscript writing. TO was responsible for animal experiments and data collection and PET data analysis. BH and MF were responsible for μCT data analysis and interpretation. HS and TK were responsible for data interpretation and critical revision of manuscript. All authors read and approved the final version of the manuscript.

## Supplementary Material

Additional file 1: Figure S1 ^**18**^**F-fluoride accumulation in healthy mice.** In healthy mice, application of ^18^F-fluoride resulted in distinct bone tracer accumulation in the spine, skull, pelvis, pectoral girdle, elbow and knee joints. **(A)** Coregistration of PET and CT data revealing exact sites of ^18^F-fluoride accumulation, and PET imaging of ^18^F-fluoride signaling 90 minutes after radiotracer injection in a naïve mouse. **(B)** Coronal and transverse views of the pelvis–knee region showing ^18^F-fluoride accumulation in trabecular bone. **(C)** Visualization of increased PET signaling in the knee joint (red) by volume rendering and trabecular structure of bone (right). Click here for file
