# Supplementary figures and images for: 18 F-Fluoride positron emission tomography/computed tomography for noninvasive in vivo quantification of pathophysiological bone metabolism in experimental murine arthritis
Source: Arthritis Res Ther. 2014 Jul 22;16(4):R155. doi: 10.1186/ar4670 (PMC4220085; doi:10.1186/ar4670)

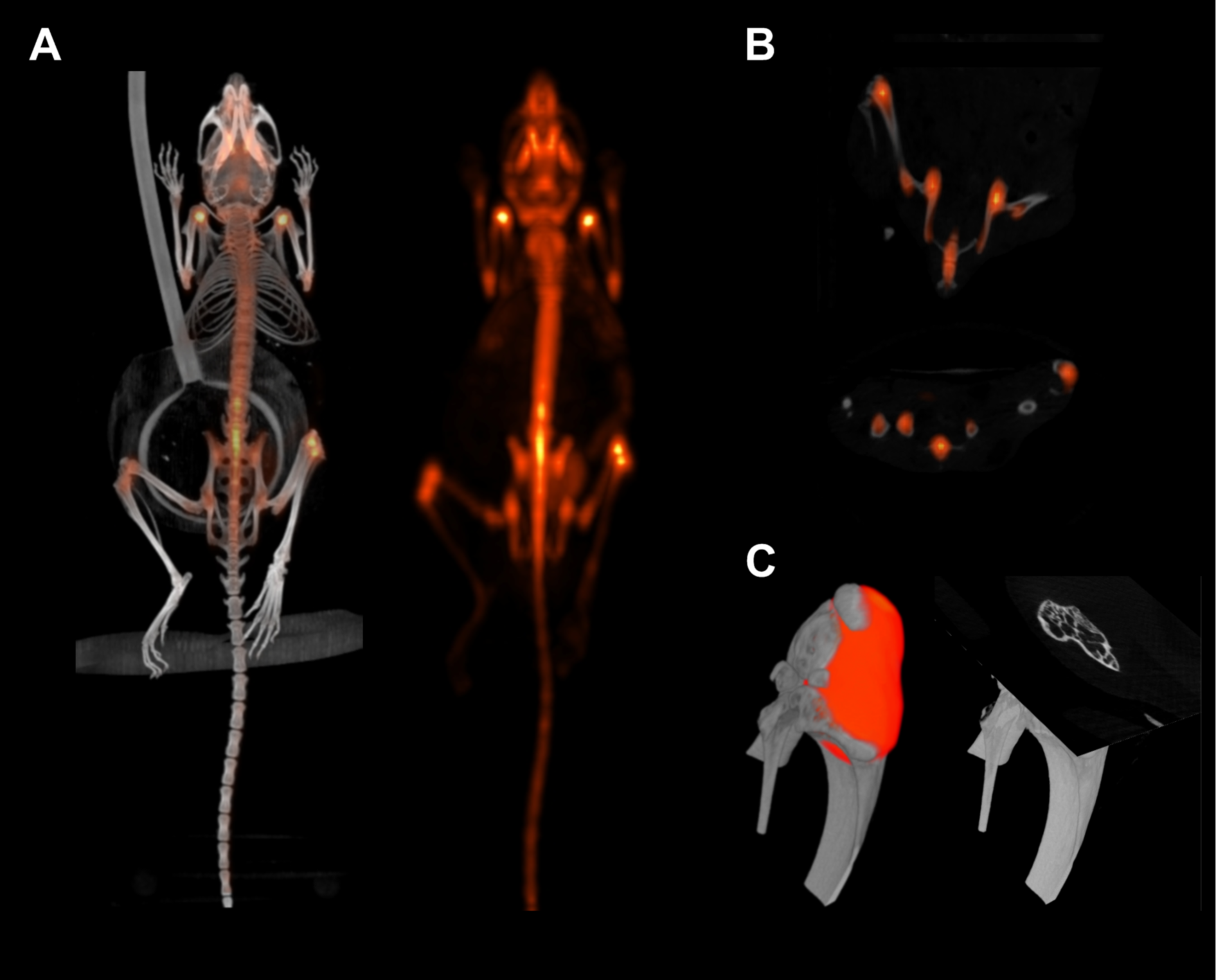

Supplement: Additional file 1: Figure S1 — 18F-fluoride accumulation in healthy mice. In healthy mice, application of 18F-fluoride resulted in distinct bone tracer accumulation in the spine, skull, pelvis, pectoral girdle, elbow and knee joints. (A) Coregistration of PET and CT data revealing exact sites of 18F-fluoride accumulation, and PET imaging of 18F-fluoride signaling 90 minutes after radiotracer injection in a naïve mouse. (B) Coronal and transverse views of the pelvis–knee region showing 18F-fluoride accumulation in trabecular bone. (C) Visualization of increased PET signaling in the knee joint (red) by volume rendering and trabecular structure of bone (right). [file ar4670-S1.tiff]
